# Plasma Exosome-Derived Sentrin SUMO-Specific Protease 1: A Prognostic Biomarker in Patients With Osteosarcoma

**DOI:** 10.3389/fonc.2021.625109

**Published:** 2021-03-09

**Authors:** Li Wang, Jian Wu, Shu Song, Haining Chen, Yong Hu, Buwei Xu, Jinbo Liu

**Affiliations:** ^1^ Department of Orthopedics, The Third People’s Hospital of Yancheng City, Yancheng, China; ^2^ Department of Laboratory Medicine, The First People’s Hospital of Yancheng City, Yancheng, China; ^3^ Department of Pathology, Shanghai Public Health Clinical Center, Fudan University, Shanghai, China; ^4^ Department of Orthopedics, The Affiliated Hospital of Jiangsu University, Zhenjiang, China; ^5^ Department of Orthopedics, The Third Affiliated Hospital of Soochow University, Changzhou, China; ^6^ Department of Spine, The Third Affiliated Hospital of Soochow University, Changzhou, China

**Keywords:** sentrin sumo-specific protease 1 (SENP1), osteosarcoma, exosomes, prognostic biomarker, small ubiquitin-related modifier (SUMO)

## Abstract

**Background:**

The exosomes contain many important proteins that can be used for early tumor diagnosis or patient prognosis analysis. In this study, we investigated plasma exosome-derived sentrin SUMO-specific protease 1 (SENP1) levels as a prognostic biomarker in patients with osteosarcoma.

**Methods:**

The expression of SENP1 protein in osteosarcoma tissues and adjacent tissues was detected by immunohistochemistry (IHC). The exosomes were identified by transmission electron microscopy, nanoparticle tracking analysis, and western blotting. ELISA was used to detect plasma exosome-derived SENP1 levels to assess prognosis in patients with osteosarcoma.

**Results:**

IHC showed that the positive expression rate of SENP1 in osteosarcoma tissues was 88.33%, whereas that in adjacent tissues was 46.67% (*P* < 0.05). Plasma exosome-derived SENP1 levels were related to tumor size, tumor location, necrosis rate, pulmonary metastasis, and surgical stage. Both disease-free survival (DFS) and overall survival (OS) were worse in patients who had higher plasma exosome-derived SENP1 levels compared with those in patients with lower plasma exosome-derived SENP1 levels (*P* < 0.001). The area under the receiver operating characteristic curve (AUROC) of plasma exosome-derived SENP1, as 1-year DFS and 3-year DFS prognostic biomarkers, was 0.90 (95% CI: 0.83–0.98) and 0.96 (95% CI: 0.94–0.99), respectively. As to OS, the AUROC of plasma exosome-derived SENP1 for 1-year and 3-year prediction was 0.90 (95% CI: 0.82–0.99) and 0.96 (0.93–0.98), respectively. The plasma exosome-derived SENP1 was better than plasma SENP1 as a prognostic biomarker both in DFS and OS.

**Conclusions:**

Our findings show that the plasma exosome-derived SENP1 may serve as a novel and independent prognostic predictor in clinical applications.

## Introduction

Osteosarcoma is a malignant tumor that often occurs in adolescents and mostly grows around the knee joint. Many studies have shown that the worldwide incidence of osteosarcoma is about 0.0004% (1, 2). The early symptoms of osteosarcoma are mainly swelling and pain at the site of the disease, which are not highly specific and can easily lead to clinical misdiagnosis and missed diagnosis (3). Common complications of osteosarcoma are lung metastasis and pathological fracture. Numerous studies have proved that most patients have distant metastases when they are diagnosed, so they have poor prognosis (4, 5). There are many different treatment methods for osteosarcoma including surgery and chemotherapy. Although the survival time of patients without metastasis increased by about 70%, patients in remission after clinical treatment also have a high risk of recurrence, because of the high degree of malignancy, aggressiveness, and rapid progression of osteosarcoma (6). There are still a lack of tumor markers for early diagnosis and prognostic evaluation of osteosarcoma (7). Hence, it is important to find clinically specific, conveniently detected and effective biomarkers for early diagnosis and prognostic evaluation of osteosarcoma.

Small ubiquitin-related modifier (SUMO) is a small ubiquitin-like protein with a molecular weight of 12 kDa and consisted of about 100 amino acids (8). The modification of SUMO is mainly catalyzed by SUMO-specific protease (SENP), conjugating enzyme E2 (Ubc9) and E3 ligase (9). SUMO modification participates in the basic physiological activities of cells, which is one of the necessary mechanisms for growth and development. SUMO is also involved in cellular stress, including exogenous and endogenous injury stress (10, 11). SUMO modification participates in various physiological activities of cells by regulating the function of its target protein. SENP1 plays an important role both in maturation and depolymerization of SUMO (12, 13). In recent years, it has been found that SENP1 is highly expressed in prostate, pancreatic and colon cancer, and knockout of SENP1 greatly affects the biological function of these tumors (14–16). Wang et al. have revealed that the expression of SENP1 is also markedly upregulated in osteosarcoma cells (17).

Exosomes are released after cell membrane fusion and their size ranges from 30 to 150 nm (18, 19). Exosomes are vesicle-like structures that can be widely found in blood, dendritic, tumor, and other types of cells, and can be released under both physiological and pathological conditions (20). Extracellular vesicles contain receptors, bioactive lipids, proteins, and nucleic acids and play an important role in cell-to-cell communication (21). A large number of studies (22, 23) have reported that the exosomes contain many important proteins that can be used for early tumor diagnosis or patient prognosis analysis. Hence, in this study we investigated the prognostic value of SENP1 in patients with osteosarcoma by determining the concentration of plasma exosomes from patients.

## Materials and Methods

### Patients and Clinical Samples

We collected peripheral blood samples from 146 patients with osteosarcoma who underwent surgical resection at Yancheng Clinical Medical College of Nanjing Medical University, Shanghai Public Health Clinical Center, the Affiliated Hospital of Jiangsu University, the Third Affiliated Hospital of Soochow University, and the First People’s Hospital of Yancheng City, between January 2017 and December 2019. Among the enrolled 146 osteosarcoma patients, the tumor tissues and paired adjacent tissues of 60 cases were isolated during surgery used to detect SENP1 expression by immunohistochemistry (IHC). Tissue sections from all enrolled patients were confirmed independently by two pathologists. The diagnostic criteria were based on the American Joint Committee on Cancer Staging System (AJCC) (24).

The blood samples from each patient were processed within 30 min after collection, and centrifuged for 15 min at 3,000×g at 4°C and stored at −80°C. We collected relevant clinical data from patients, including age, sex, depth of invasion, tumor size, lymph node metastasis, AJCC stage, and pathological differentiation. Patients were followed up to June 30, 2020, with a median follow-up duration of 27.5 months (range: 6.0–38.0 months). The survival data were collected from follow-up records, and disease-free survival (DFS) and overall survival (OS) were calculated. DFS was defined as the duration from resection to disease recurrence, disease progression, or death. OS was defined as the time interval from resection to death. The follow-up results for the 146 patients were obtained by medical records and telephone interviews. The study was approved by the Ethics Committees of Yancheng Clinical Medical College of Nanjing Medical University [identification nos. HMU (Ethics) 2017-k-133].

### IHC

IHC was performed to detect the distribution of SENP1 expression. An Envision and DAB Chromogenic Reagent Kit (Antibody Diagnostic Inc., USA) was used for IHC. Paraffin-embedded specimens were dewaxed in xylene and a graded series of ethanol (absolute, 95%, 85%, and 75%), and antigen retrieval was performed using citrate buffer. Specimens were incubated with 10% goat serum at room temperature for 30 min. The primary antibody to SENP1 (1: 250, Abcam, Cambridge, UK) was incubated with the sections for 1 h at room temperature. Sections were incubated with secondary antibodies at room temperature for 1 h and washed with diaminobenzidine (DAB) (Boster Bio, Pleasanton, CA, USA)  at room temperature for 5 min. Counterstaining was performed using hematoxylin with 1% hydrochloric acid and ammonia water anti-blue for 20 s. The positive area was observed under an optical microscope, and the proportion of the positive area was calculated. IHC results were assessed by two pathologists, and positively stained cells in osteosarcoma and adjacent tissues were observed. Each section was randomly selected with 10 high-power fields, and 100 tumor cells were counted in each field. According to the degree of staining and the number of positive cells to calculate the score (1). Staining intensity: 0 points, negative; 1 point, weak positive; 2 points, positive; and 3 points, strong positive (2). The number of positive cells: 0 points, no positive cells; 1 point, 1–25% positive cells; 2 points, 26–50% positive cells; and 3 points, >50% positive cells. If the sum of the two scores was 3–6 points, they were rated as positive.

### Plasma Exosomes Isolation

Human plasma (1–2 ml) (diluted five times in 1×PBS) was centrifuged at 500×g at 4°C for 5 min to remove cells. The supernatant was centrifuged at 2,000×g at 4°C for 10 min to remove cell debris. After centrifugation at 10,000×g at 4°C for 30 min, large vesicles were removed. The supernatant was filtered with a 0.22-μm filter to remove any large particles. Take the filtered supernatant to the ultracentrifugation tube, fill up the remaining volume with 1×PBS buffer solution, accurately weigh and balance, put it on the rotor of the ultracentrifuge, centrifuge at 100,000×g, 4°C, and take the filtered supernatant the human plasma sample for 2 h. After centrifugation, the supernatant was discarded and a translucent sediment was seen at the bottom of the tube. The sediment was resuspended in 1×PBS buffer and centrifuged at 100,000×g at 4°C for 80 min. The exosomes were resuspended with 100–200 μl 1×PBS buffer and transferred to 1.5-ml Eppendorf tube. According to the requirements of follow-up experiments, downstream experiments can be carried out directly and stored at −80°C.

### Transmission Electron Microscopy

After centrifugation, the exosome precipitate was resuspended with 100 μl PBS. A heavy suspension of 20 μl was loaded on a copper wire mesh. The sample was left standing for 2 min at room temperature. The liquid was carefully absorbed from the side of the filter screen with filter paper. 3% phosphotungstic acid solution (20 μl) was dripped into the solution and dyed 3% at room temperature. The copper mesh was washed five times with double-distilled water. After natural drying at room temperature, the samples were observed and photographed under a transmission electron microscope (Thermo-Fisher, Waltham, MA, USA).

### Nanoparticle Tracking Analysis

The exosome suspension was blown evenly with a pipette, diluted 400–1000 times with 1×PBS filtered at 0.22 μm, adjusted to the optimal detection concentration (20–100 particles per field) of NanoSight NS300, and 1 ml was injected into the instrument. A laser (blue 488 nm) was used to irradiate the sample, and the average frame rate of 20 frames per second was used to record the movement of nanoparticles due to Brownian motion for 1 min. Each process was repeated three times. Finally, the data were output and analyzed by NTA 3.3 software to obtain the size distribution and particle concentration of exosomes.

### Western Blotting

Exosomes were isolated and added to sodium dodecyl sulfate (SDS) buffer for total proteins. Total protein was separated by SDS-PAGE and transferred onto polyvinylidene difluoride membranes (Millipore, Billerica, MA, USA). After blocking in 5% non-fat milk for 1 h, membranes were incubated overnight at 4°C with the indicated primary antibodies, including Annexin V, TSG101, CD9, and CD63 (Santa Cruz Biotechnology, Dallas, TX, USA), followed by incubation with secondary antibodies for 1 h at room temperature.

### ELISA

The plasma sample with residual cells and cell fragments removed was diluted with 1×PBS (1:500 dilution). The exosomes were precipitated with 100 ml RIPA lysate on ice for 30 min. After shaking and mixing, the samples were diluted with PBS (1:3 dilution). The standard substance and blank control were added to the microplate coated with SENP1 antibody. The diluted exosome samples were 100 μl. Incubate at 37°C for 60 min, shake off the liquid in the microplate, pat dry, add liquid a, incubate at 37°C for 60 min, wash the plate for 3 times, add liquid B, incubate at 37°C for 30 min, wash the plate for 5 times, add 90 μl substrate, incubate for 15 min at 37°C away from light, and add 50 μl of termination solution, and measure at 450 nm wavelength immediately. At the same time, the above methods were used to detect the level of plasma SENP1 protein, which was used as a control group.

### Statistical Analysis

Statistical analyses were performed using SPSS 24.0 software (IBM, Armok, NY, USA), and figures were plotted using GraphPad Prism 7.00 (GraphPad Software, La Jolla, CA, USA). Data were presented as mean ± standard deviation, median (range), or count (percentage). The expression of SENP1 in tumor tissue and paired adjacent tissue was compared using the chi square test. Correlation analyses were performed using the Mann–Whitney test. The prognostic value of plasma exosome-derived SENP1 levels in osteosarcoma was assessed using receiver operating characteristic (ROC) curve analysis. Results with *P* < 0.05 were considered significant.

## Results

### Baseline Characteristics

**Table 1** shows the baseline characteristics of all enrolled 146 osteosarcoma patients: 88 (60.27%) were men and 58 (39.73%) were women. Ninety-four patients (64.38%) were aged <20 years, and 52 patients (35.62%) were >20 years. Tumor size was <10 cm in 75 patients, and >10 cm in 71. The tumor location in 89 patients (60.96%) was in the proximal extremity, and in the distal extremity in the other 57 patients (39.04%). Sixty patients (41.10%) had pulmonary metastasis, and 56 (38.36%) had a necrosis rate >90%. In addition, 82 (56.16%) and 64 (43.84%) patients had AJCC stages I+II, and III +IV, respectively.

**Table 1:** Baseline characteristics of enrolled osteosarcoma patients.

Characteristic	Osteosarcoma patients (n = 146)
**Gender**	
Male	88 (60.27)
Female	58 (39.73)
**Age (years)**	
<20	94 (64.38)
≥20	52 (35.62)
**Tumor diameter (cm)**	
<10	75 (51.37)
≥10	71 (48.63)
**Tumor location**	
Proximal extremity	89 (60.96)
Distal extremity	57 (39.04)
**Necrosis rate**	
<90%	90 (61.64)
≥90%	56 (38.36)
**Pulmonary metastasis**	
YES	60 (41.10)
NO	86 (58.90)
**Tumor stage (AJCC)**	
I+II	82 (56.16)
III +IV	64 (43.84)

AJCC, American Joint Committee on Cancer Staging System.

### SENP1 Expression in Osteosarcoma and Adjacent Tissues

**Figure 1:**
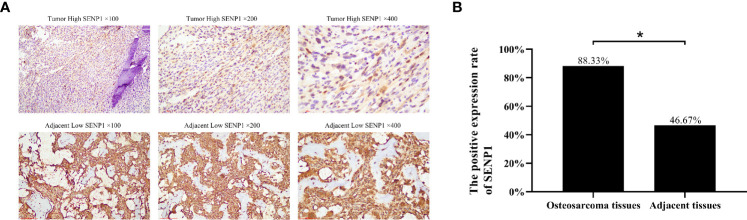
Immunohistochemical analysis of SENP1 expression in osteosarcoma tissues and adjacent tissues. **(A)** High SENP1 expression in osteosarcoma tissues at 100×, 200× and 400× magnification. Low SENP1 expression in adjacent tissues at 100×, 200× and 400× magnification; **(B)** the positive rate of SENP1 protein expression in osteosarcoma tissues and adjacent tissues. (*P < 0.05).

The expression of SENP1 in osteosarcoma and adjacent tissues was evaluated by IHC. SENP1 expression in osteosarcoma tissues was significantly higher than in adjacent tissues (**Figure 1A**). The positive expression rate of SENP1 in osteosarcoma tissues was 88.33% (53/60), whereas that in adjacent tissues was 46.67% (28/60) (*P* < 0.05; **Figure 1B**).

### Characterization of Exosomes Isolated From Plasma

**Figure 2:**
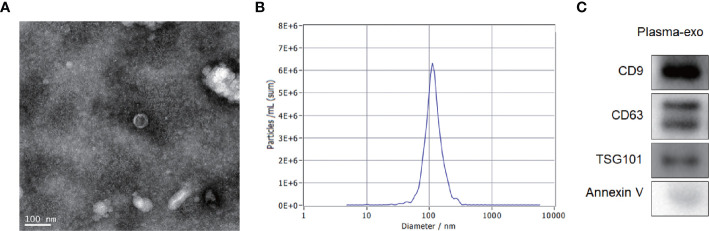
Patient exosome characterization. **(A)** TEM images showed typical exosomes with oval or bowl-shaped microvesicles. **(B)** NTA data revealed that patient plasma exosome peak sizes were 50–120 nm. **(C)** Western blotting showed that patient plasma exosomes were positive for the four exosomal markers, annexin V, Tsg101, CD9, and CD63.

The exosome integrity and purification were confirmed with TEM and, NTA and four antibody markers for extracellular vesicles (western blot analysis). The exosomes obtained by low temperature gradient ultracentrifugation were fixed and stained. TEM images showed typical exosomes with oval or bowl-shaped microvesicles (**Figure 2A**). The 400-fold diluted exosomes were resuspended with filtered PBS and injected into NanoSight NS300. The NTA data revealed that patient plasma exosome peak sizes were 50–120 nm (**Figure 2B**). Western blotting showed that patient plasma exosomes were positive for the four exosomal markers, including Annexin V, Tsg101, CD9, and CD63 (**Figure 2C**).

### Relationship Between Plasma Exosome-Derived SENP1 Levels and Tumor Characteristics

**Figure 3:**
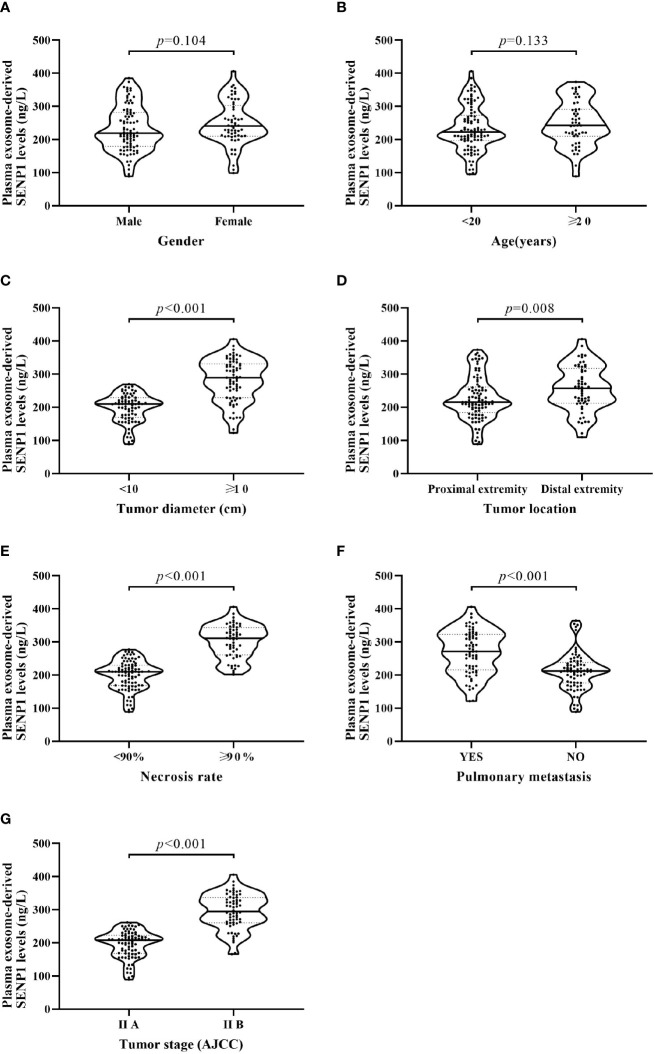
Relationship between plasma exosome-derived SENP1 levels and tumor characteristics. **(A)** No significant differences were observed between male and female patients (*P* = 0.104). **(B)** No significant differences were found between patients aged <20 and >20 years (*P* = 0.133). **(C)** Plasma exosome-derived SENP1 levels of patients with osteosarcoma with tumor size >10 cm were significantly higher than those in patients with tumor size <10 cm (*P* < 0.001). **(D)** Plasma exosome-derived SENP1 levels of patients with osteosarcoma located in distal extremity were significantly higher than those in patients with osteosarcoma located in proximal extremity (*P* = 0.008). **(E)** Plasma exosome-derived SENP1 levels of patients with osteosarcoma with necrosis rate >90% were significantly higher than those in patients with necrosis rate <90% (*P* < 0.001). **(F)** Plasma exosome-derived SENP1 levels of patients with osteosarcoma with pulmonary metastasis were significantly higher than those in patients without pulmonary metastasis (*P* < 0.001). **(G)** Plasma exosome-derived SENP1 levels of patients with osteosarcoma in surgical stag III;+IV were significantly higher than those in patients with osteosarcoma in surgical stage I+II (*P* < 0.001).

**Figure 4:**
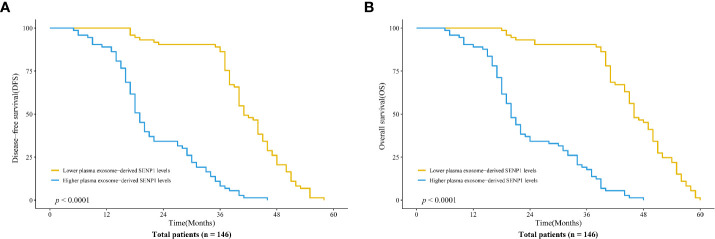
Association of plasma exosome-derived SENP1 levels with disease-free survival (DFS) and overall survival (OS) in patients with osteosarcoma. **(A)** Among all patients with osteosarcoma, DFS was worse in patients who had higher compared with lower plasma exosome-derived SENP1 levels (*P* < 0.001). **(B)** Among all patients with osteosarcoma, OS was worse in patients who had higher compared with lower plasma exosome-derived SENP1 levels (*P* < 0.001).

We assessed the correlations between plasma exosome-derived SENP1 levels and tumor characteristics in patients with osteosarcoma. According to sex and age, there was no significant difference in the plasma exosome-derived SENP1 levels (*P* > 0.05, **Figures 3A, B**). However, the plasma exosome-derived SENP1 levels of patients with osteosarcoma with tumor size >10 cm, located in the distal extremity, necrosis rate >90%, pulmonary metastasis, and surgical stage III; +IV were significantly higher than in patients in with tumor size <10 cm, located in the proximal extremity, necrosis rate <90%, without pulmonary metastasis, and in surgical stage ι+II (*P* < 0.05, **Figures 3C–G**). Among all patients with osteosarcoma, both DFS and OS were worse in patients who had higher plasma exosome-derived SENP1 levels compared with those in patients with lower plasma exosome-derived SENP1 levels (*P* < 0.001; **Figure 4**).

### Prognostic Value of Plasma Exosome-Derived SENP1 Levels in Osteosarcoma

**Figure 5:**
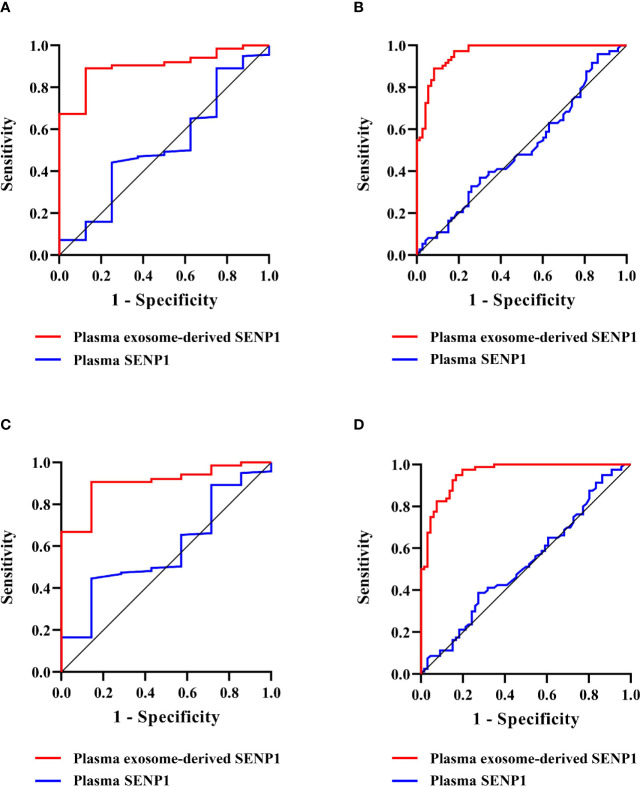
Prognostic value of plasma exosome-derived SENP1 levels in patients with osteosarcoma. **(A)** AUC for plasma exosome-derived SENP1 levels and plasma SENP1 levels for the prognostic value in patients with osteosarcoma 1-year DFS were 0.90 (95% CI: 0.83–0.98) and 0.52 (95% CI: 0.31–0.73), respectively. **(B)** AUC for plasma exosome-derived SENP1 levels and plasma SENP1 levels for the prognostic value in patients with osteosarcoma 3-year DFS were 0.96 (95% CI: 0.94–0.99) and 0.51 (95% CI: 0.41–0.60), respectively. **(C)** AUC for plasma exosome-derived SENP1 levels for the prognostic value in patients with osteosarcoma 1-year OS were 0.90 (95% CI: 0.82–0.99) and 0.59 (95% CI: 0.39–0.78), respectively. **(D)** AUC for plasma exosome-derived SENP1 levels for the prognostic value in patients with osteosarcoma 3-year OS were 0.96 (95% CI: 0.93–0.98) and 0.53 (95% CI: 0.43–0.62), respectively.

We assessed the prognostic value of plasma exosome-derived SENP1 using ROC curve analysis (**Table 2**). The area under the ROC curve (AUROC) of plasma exosome-derived SENP1, as 1-year DFS prediction biomarkers, was 0.90 (95% CI: 0.83–0.98), while the AUROC of plasma SENP1 was 0.52 (95% CI: 0.31–0.73) (**Figure 5A**). With the cutoff value of 322.17, the positive predictive value and positive likelihood ratio of plasma exosome-derived SENP1 were 31.8 (95% CI: 13.9–54.9) and 8.05 (95% CI: 4.7–13.9), respectively. The negative predictive value and negative likelihood ratio were 99.2 (95% CI: 95.6–100.0) and 0.14 (95% CI: 0.02–0.9), respectively, with a sensitivity of 87.5% (95% CI: 47.3–99.7%) and specificity of 89.1% (95% CI: 82.7–93.8%). For 3-year DFS, the AUROC of plasma exosome-derived SENP1 was 0.96 (95% CI: 0.94–0.99), while the AUROC of plasma SENP1 was 0.51 (95% CI: 0.41–0.60) (**Figure 5B**). With the cutoff value of 226.25, the positive predictive value and positive likelihood ratio of plasma exosome-derived SENP1 were 89.3 (95% CI: 80.1–95.3) and 8.38 (95% CI: 4.3–16.2) respectively. The negative predictive value and negative likelihood ratio were 91.5 (95% CI: 82.5–96.8) and 0.092 (95% CI: 0.04–0.2), respectively, with a sensitivity of 91.8% (95% CI: 83.0–96.9%) and specificity of 89.0% (95% CI: 79.5–95.1%).

**Table 2:** The prognostic value of plasma exosome-derived SENP1 levels in osteosarcoma.

Variable	(n = 146)
**AUROC (1-year DFS)**	0.90 (0.83–0.98)
**Cutoff value (95% CI)**	322.17
Sensitivity, %	87.5 (47.3–99.7)
Specificity, %	89.1 (82.7–93.8)
Positive predictive value, %	31.8 (13.9–54.9)
Negative predictive value, %	99.2 (95.6–100.0)
Positive likelihood ratio	8.05 (4.7–13.9)
Negative likelihood ratio	0.14 (0.02–0.9)
**AUROC (3-year DFS)**	0.96 (0.94–0.99)
**Cutoff value (95% CI)**	226.25
Sensitivity, %	91.8 (83.0–96.9)
Specificity, %	89.0 (79.5–95.1)
Positive predictive value, %	89.3 (80.1–95.3)
Negative predictive value, %	91.5 (82.5–96.8)
Positive likelihood ratio	8.38 (4.3–16.2)
Negative likelihood ratio	0.092 (0.04–0.2)
**AUROC (1-year OS)**	**0.90 (0.82–0.99)**
**Cutoff value (95% CI)**	327.81
Sensitivity, %	85.7 (42.1–99.6)
Specificity, %	90.6 (84.5–94.9)
Positive predictive value, %	31.6 (12.6–56.6)
Negative predictive value, %	99.2 (95.7–100.0)
Positive likelihood ratio	9.16 (5.0–16.7)
Negative likelihood ratio	0.16 (0.03–1.0)
**AUROC (3-year OS)**	**0.96 (0.93-0.98)**
**Cutoff value (95% CI)**	249.33
Sensitivity, %	83.3 (72.1–91.4)
Specificity, %	95.0 (87.7–98.6)
Positive predictive value, %	93.2 (83.5–98.1)
Negative predictive value, %	87.4 (78.5–93.5)
Positive likelihood ratio	16.67 (6.4–43.6)
Negative likelihood ratio	0.18 (0.1–0.3)

SENP1, sentrin sumo-specific protease 1; AUROC, area under the receiver operating characteristic curve; DFS, disease-free survival; OS, overall survival.

The AUROC of plasma exosome-derived SENP1 for 1-year OS prediction was 0.90 (95% CI: 0.82–0.99), while the AUROC of plasma SENP1 was 0.59 (95% CI: 0.39–0.78) (**Figure 5C**). With the cutoff value of 327.81, the positive predictive value and positive likelihood ratio of plasma exosome-derived SENP1 were 31.6 (95% CI: 12.6–56.6) and 9.16 (95% CI: 5.0–16.7), respectively. The negative predictive value and negative likelihood ratio were 99.2 (95% CI: 95.7–100.0) and 0.16 (95% CI: 0.03–1.0), respectively, with a sensitivity of 85.7% (95% CI: 42.1–99.6%) and specificity of 90.6% (95% CI: 84.5–94.9%). For 3-year OS, the AUROC of plasma exosome-derived SENP1 was 0.96 (95% CI: 0.93–0.98), while the AUROC of plasma SENP1 was 0.53 (95% CI: 0.43–0.62) (**Figure 5D**). With the cutoff value of 249.33, the positive predictive value and positive likelihood ratio of plasma exosome-derived SENP1 were 93.2 (95% CI: 83.5–98.1), and 16.67 (95% CI: 6.4–43.6), respectively. The negative predictive value and negative likelihood ratio were 87.4 (95% CI: 78.5–93.5) and 0.18 (95% CI: 0.1–0.3), respectively, with a sensitivity of 83.3% (95% CI: 72.1–91.4%) and specificity of 95.0% (95% CI: 87.7–98.6%).

## Discussion

In recent years, the early diagnosis and prognostic monitoring of tumors have received much attention (25, 26). More striking is that exosomes contain many important proteins, which can be used for early diagnosis of tumors or prognostic analysis of patients. Among them, exosome proteins isolated from plasma have become a hot topic in biomarker research. SENP1 is an important member of the enzyme protein family that regulates the reverse reaction of SUMO modification (27). Previous studies have shown that SENP1 plays an important role in the occurrence and progression of malignant tumors by regulating hypoxia-inducible factor (HIF)-1α (28, 29). SENP1 is the regulatory target gene of HIF-1α. The positive feedback between SENP1 and HIF-1α can improve the stem cell characteristics of hepatocellular carcinoma (HCC) and promote the occurrence of HCC (30). Inhibition of SENP1 expression in high-grade metastatic prostate cancer cells can affect the bone remodeling ability of HIF-1α and its downstream matrix metalloproteinase (MMP)-2 and MMP-9, and then inhibit the bone metastasis of prostate cancer cells (31).

Dong et al. revealed that SENP1 promotes the proliferation of renal clear cell carcinoma by activating glycolysis (32). Liu et al. demonstrated that the high expression of SENP1 protein in non-small cell lung cancer (NSCLC) tissues, and SENP1 protein is a risk factor for poor prognosis of NSCLC (33). Cai et al. showed that the variation of SENP1 in the Chinese population is not related to the risk of breast cancer, but has a specific impact on the clinicopathological characteristics of breast cancer. SENP1 rs61918808 may be a predictor of clinical response in locally advanced breast cancer patients receiving neoadjuvant chemotherapy (34). SENP1 expression has also been detected in osteosarcoma cells. SENP1 promotes proliferation, invasion, and epithelial–mesenchymal transition of osteosarcoma cells by regulating HIF-1α expression under hypoxia (35). However, the expression and prognostic value of SENP1 in patients with osteosarcoma remain unclear. To date, this is the first study to examine the potential of plasma exosome-derived SENP1 in osteosarcoma diagnosis.

In this study, we demonstrated using IHC that the expression of SENP1 in osteosarcoma tissues was significantly higher than that in adjacent tissues, which is consistent with Huang et al. (35). In order to study the role of plasma exosome-derived SENP1 in patients with osteosarcoma, we extracted exosomes from the plasma of patients with osteosarcoma. TEM images showed typical exosomes with oval or bowl-shaped microvesicles. The NTA data revealed that patient plasma exosome peak sizes were 50–120 nm. Although the components of exosomes vary according to different cell and tissue sources, all exosomes contain membrane transporters and fusion proteins (GTPases, annexins, and flotillin, etc.); heat shock proteins (HSP70, HSP90, etc.); four transmembrane proteins (CD9, CD63, CD81, and CD82); proteins involved in the biosynthesis of polyvesicles (Alix and TSG101, etc.); and lipid-related proteins. In our study, annexin V, TSG101, and CD63 were selected as the identification indexes of plasma exosomes. Western blotting showed that patient plasma exosomes were positive for the four exosomal markers, annexin V, Tsg101, CD9, and CD63.

Correlations between plasma exosome-derived SENP1 levels and tumor characteristics in patients with osteosarcoma were assessed, which showed that the plasma exosome-derived SENP1 levels were related to tumor size, tumor location, necrosis rate, pulmonary metastasis, and surgical stage. Both DFS and OS were worse in patients who had higher plasma exosome-derived SENP1 levels compared with patients with lower plasma exosome-derived SENP1 levels. Finally, we assessed the prognostic value of plasma exosome-derived SENP1 levels in osteosarcoma. Whether 1-year DFS and 3-year DFS or 1- and 3-year OS, the performance of plasma exosome-derived SENP1 was better than plasma SENP1 as a prognostic biomarker both for DFS and OS.

This study had several limitations that should be noted. Firstly, although this was the largest study to date evaluating SENP1 protein expression in osteosarcoma, detection of gene levels is necessary to confirm the findings. Secondly, the application of plasma exosome-derived SENP1 levels as a clinical prognostic biomarker in patients with osteosarcoma needs to be confirmed and validated with a large multicenter sample. Thirdly, we did not perform an assessment of the mechanisms of SENP1 function, including up- and downstream genes in osteosarcoma.

## Conclusion

In summary, our study revealed that osteosarcoma patients with higher plasma exosome-derived SENP1 levels had worse DFS and OS. Our findings may facilitate the establishment of plasma exosome-derived SENP1 levels as a novel and independent prognostic predictor in clinical applications.

## Data Availability Statement

The original contributions presented in the study are included in the article/supplementary material. Further inquiries can be directed to the corresponding authors.

## Ethics Statement

The studies involving human participants were reviewed and approved. The study was approved by the Ethics Committees of Yancheng Clinical Medical College of Nanjing Medical University (identification nos. HMU [Ethics] 2017-k-133). The consent from study participants was obtained by written or verbal. The patients/participants provided their written informed consent to participate in this study.

## Author Contributions

LW and JW contributed to study concept and design, acquisition of data, analysis and interpretation of data, and drafting of the manuscript. HC and YH contributed to study concept, study supervision, and critical revision of the manuscript. BX contributed to study supervision and critical revision of the manuscript. SS contributed to statistical analysis. JL contributed to study concept and design, study supervision, and critical revision of the manuscript. All authors contributed to the article and approved the submitted version.

## Funding

JW is recipient of fellowships from Jiangsu Young Medical Talents Project (No: QNRC00475). JW contributed to study concept and design, acquisition of data, analysis and interpretation of data, and drafting of the manuscript.

## Conflict of Interest

The authors declare that the research was conducted in the absence of any commercial or financial relationships that could be construed as a potential conflict of interest.

## Abbreviations

SENP1, sentrin sumo-specific protease 1; SUMO, small ubiquitin-related modifier; IHC, immunohistochemistry; NTA, nanoparticle tracking analysis; TEM, transmission electron microscopy; DFS, disease-free survival; OS, overall survival.
